# Comparative structural analysis of the caspase family with other clan CD cysteine peptidases

**DOI:** 10.1042/BJ20141324

**Published:** 2015-02-20

**Authors:** Karen McLuskey, Jeremy C. Mottram

**Affiliations:** *Wellcome Trust Centre for Molecular Parasitology, Institute of Infection, Immunity and Inflammation, College of Medical, Veterinary and Life Sciences, University of Glasgow, Glasgow G12 8TA, UK

**Keywords:** caspase, clan CD, crystallography, metacaspase, peptidase, protein structure, AP, activation peptide, CARD, caspase recruitment domain, CHF, caspase/haemoglobinase fold, CPD, cysteine peptidase domain, CSD, C-terminal subdomain, DD, death domain, DED, death effector domain, Ins*P*_6_, *myo*-inositol hexakisphosphate, LSAM, legumain stabilization and activity modulation, LSD1, lesion-simulating disease 1, MALT1, mucosa-associated lymphoid tissue translocation protein 1, MARTX, multi-functional, autoprocessing repeat in toxin, RMSD, root-mean-square deviation, SSE, secondary structural element, XIAP, X-linked inhibitor of apoptosis, Z-VRPR-FMK, benzoxycarbonyl-Val-Arg-Pro-Arg-fluoromethylketone

## Abstract

Clan CD forms a structural group of cysteine peptidases, containing seven individual families and two subfamilies of structurally related enzymes. Historically, it is most notable for containing the mammalian caspases, on which the structures of the clan were founded. Interestingly, the caspase family is split into two subfamilies: the caspases, and a second subfamily containing both the paracaspases and the metacaspases. Structural data are now available for both the paracaspases and the metacaspases, allowing a comprehensive structural analysis of the entire caspase family. In addition, a relative plethora of structural data has recently become available for many of the other families in the clan, allowing both the structures and the structure–function relationships of clan CD to be fully explored. The present review compares the enzymes in the caspase subfamilies with each other, together with a comprehensive comparison of all the structural families in clan CD. This reveals a diverse group of structures with highly conserved structural elements that provide the peptidases with a variety of substrate specificities and activation mechanisms. It also reveals conserved structural elements involved in substrate binding, and potential autoinhibitory functions, throughout the clan, and confirms that the metacaspases are structurally diverse from the caspases (and paracaspases), suggesting that they should form a distinct family of clan CD peptidases.

## INTRODUCTION

Clan CD [1] cysteine peptidases use an active site cysteine residue to catalyse the hydrolysis, and subsequent cleavage, of peptide bonds in proteins. These peptidases generally show a strict specificity for the P_1_ residue of their substrates and depend only on two key catalytic residues: a highly conserved histidine/cysteine dyad. In clan CD, the order, type and surrounding tertiary structure of the dyad are used to assign enzymes to the clan [2].

The founding member of clan CD was discovered in 1994 by the determination of the 3D crystal structure of human interleukin-1β-converting enzyme [3,4], also known as caspase-1. This structure revealed a novel protein fold and was consequently placed in a new structural group of cysteine peptidases (clan CD [5]) as family C14, which is often referred to as the caspase family. Since its discovery, clan CD has been expanded to include seven peptidase families: clostripains (C11); legumains (C13); caspases (C14); gingipains (C25); separases (C50); the cysteine peptidase domain (CPD) of the multi-functional, autoprocessing repeats in toxin (MARTX) toxins (C80); and most recently the enzymes related to the peptidase virulence factor PrtH from *Tannerella forsythia* [6] (C85). Notably, family C14 is further divided to contain subfamilies C14A (the caspases) and C14B {both the metacaspases and the paracaspases [denoted C14B(M) and C14B(P), respectively]}. The phylogenetic distribution of the clan CD peptidases spans all the kingdoms of life (Table 1). However, the caspase family (C14) is the only family that has been identified in all kingdoms, although each subfamily is found only in certain branches [7] (Table 1).

**Table 1:** The structural availability and phylogenetic distribution of the clan CD families The availability (✓) and absence (×) of clan CD families in the phylogenetic kingdom. The year that the first structure became available is shown for each family (year).

FAMILY	Representative member	Structural data? (year)	Bacteria	Achaea	Protozoa	Fungi	Plants	Viruses	Animals
C11	Clostripain	Yes (2013)	✓	✓	✓	×	✓	×	×
C13	Legumain	Yes (2013)	✓	✓	✓	✓	✓	×	✓
C14A	Caspase	Yes (1994)	×	×	×	×	×	✓	✓
C14B(P)	Paracaspase	Yes (2011)	✓	✓	×	×	×	×	✓
C14B(M)	Metacaspase	Yes (2012)	✓	✓	✓	✓	✓	×	×
C25	Gingipain R	Yes (1999)	✓	✓	×	×	×	×	×
C50	Separase	No	×	×	✓	✓	✓	×	✓
C80	MARTX-CPD	Yes (2008)	✓	×	×	×	×	×	✓
C84	PrtH peptidase	No	✓	×	×	×	×	×	×
Clan CD			✓	✓	✓	✓	✓	✓	✓

Since the structural determination of caspase-1, approximately 170 caspase structures have been deposited in the Protein Data Bank (PDB [8], www.rcsb.org) with around 30 of them assumed to be unique (<90% sequence identity). In addition, X-ray crystal structures are also available for families C11 (unpublished, PDB ID 3UWS), C13 [9], C14B(P) [10], C14B(M) [11,12], C25 [13,14] and C80 [15–17]. Apart from the original structure of gingipain R [13] (RgpB, C25), all these structures have been determined within the last 6 years (Table 1), making it an exciting time to analyse this diverse and structurally expanding clan. The present review summarizes the collective structural information of the families, evaluates and compares the structure–function relationships, and allows for greater understanding of the enzymes in clan CD.

## FAMILY C14: CASPASES, METACASPASES AND PARACASPASES

### Caspases

The name caspase is an abbreviation of **c**ysteine-dependent, **asp**artate-specific peptid**ase**, because caspases have a dominant specificity for protein substrates that contain an aspartate in the P_1_ position (Table 2). Functionally, the caspases are major regulators of apoptotic cell death pathways, proliferation and inflammation, playing vital roles in the life and death of animal cells. In humans, 11 caspases have been identified (caspase-1 to caspase-10 and caspase-14) and can be grouped together according to their sequence similarities [18], which are generally associated with their involvement in specific cellular processes [19]. They can (perhaps oversimply) be classified as either inflammatory (caspase-1, -4 and -5) or apoptotic caspases, with the latter being further organized into initiator (caspase-2, -8, -9 and -10) and effector (or executioner; caspase-3, -6 and -7) caspases [20]. Typically, caspases are described as having an N-terminal prodomain, which contains an aspartate site for (auto)proteolysis and varies in length depending on the type of caspase. The effector caspases have short prodomains (approximately 25 residues), whereas both the inflammatory and the initiator caspases have long prodomains (approximately 100–200 residues), which contain either CARD (caspase recruitment domain–inflammatory and initiator caspases) or DED (death effector domain–initiator caspases) motifs [19].

**Table 2:** Enzymatic properties of the clan CD peptidases

Family	Representative member	Specificity in P_1_	Requirement for activation	Self-inhibition observed?	Region of self-inhibition
C11	Clostripain	Arginine	Ca^2+^	Unknown	–
C13	Legumain	Asparagine and aspartate*	Change in pH	Yes	C-terminal domain
C14A	Caspase	Aspartate	Dimerization or proteolysis†	Unclear‡	N-terminal region
C14B(P)	Paracaspase	Arginine	Dimerization	Yes	Substrate-binding loop (L5)
C14B(M)	Metacaspase	Arginine and lysine§	Ca^2+^ and/or proteolysis║	Yes	N-terminal region
C25	Gingipain R	Arginine or lysine¶	Proteolysis and/or Ca^2+^**	Yes	N-terminal prodomain
C50	Separase	Arginine	Ca^2+^	Unknown	–
C80	MARTX-CPD	Leucine	Ligand binding	Yes	N-terminal region
C84	PrtH peptidase	Arginine	Unknown	Unknown	–

*Legumain will accept asparagine or aspartate residues depending on the pH.

**Cleavage of the proform of gingipain is required for full activation of the enzyme and while Ca^2+^ is not reported as a prerequisite for activation, all active forms appear to have Ca^2+^ present.

†Caspases are activated by dimerization or proteolysis depending on the type; typically initiator caspases are activated by dimerization whereas the effector caspases are activated by cleavage (proteolysis).

‡Self-inhibition using the N-terminal region has been suggested in the effector caspases but there are no structural data to date.

§Metacaspases are known to accept arginine and lysine in P_1_.

║Type I metacaspases generally activated by Ca^2+^; this is also true for type II metacaspases but, in addition, proteolysis has also been shown to be important in some cases.

¶Gingipain will accept arginine or lysine depending on the enzyme (gingipain R and K have a strict specificities for arginine and lysine, respectively).

**Figure 1:**
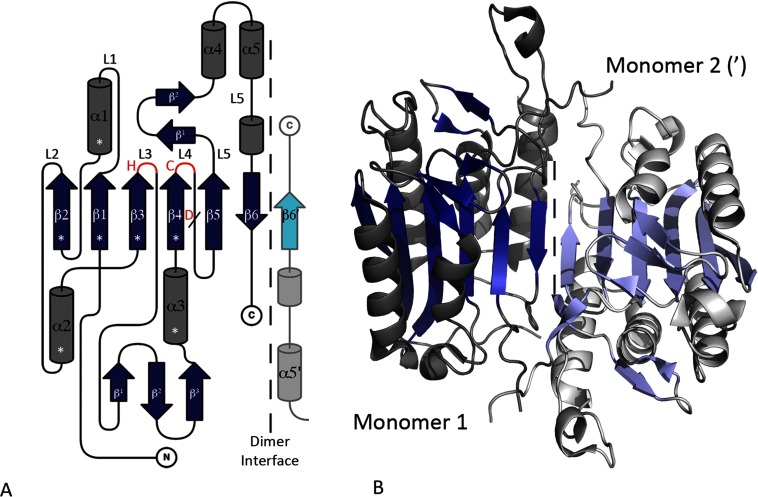
The topology and structure of the caspase dimer Caspase-7 (PDB ID 1F1J, see Supplementary Table S1) is used to represent a typical caspase with all β-strands coloured blue and α-helices grey. Structural elements from the second monomer in the dimer are coloured paler than the first. (**A**) The topology and simplified nomenclature of the caspases. The central β-strands and major α-helices are named from the N-terminus (β1–β6 and α1–α5, respectively); the important loop regions (L) are named according to the strands that they follow (L1–L5); and the small sections of β-turns on L3 and L5 are named β^1^–β^3^ and β^1^–β^2^, respectively. The position of the catalytic dyad histidine (H) and cysteine (C) is highlighted in red on L3 and L4, respectively, and the position of the conserved aspartate cleavage site (D) is shown on L4 (/). A vertical dashed line represents the dimer interface and the C-terminus of the second monomer is shown (′). The CHF SSEs are highlighted (*). (**B**) Ribbon diagram of a caspase dimer. Topdraw [90] and PyMOL (http://pymol.sourceforge.net, Schrodinger) were used for topology diagrams and molecular images throughout the present review, respectively.

In contrast to their diverse N-terminal regions, the catalytic domain of the caspases has a virtually identical fold in all the crystal structures determined to date. However, in order to describe the structure of the caspases in detail, the well-studied effector caspase, caspase-7 [21], has been chosen as a general representative of the caspases in the present review. The structure of the caspases is formed around a central six-stranded β-sheet (β1–β6), consisting of five parallel and one antiparallel β-strand(s) with 2_↑_1_↑_3_↑_4_↑_5_↑_6_↓_ topology [22]_._ The central sheet is surrounded by five major α-helices (α1–α5), contains a small three-stranded section of β-sheet situated between β3 and α3, and the residues constituting the catalytic histidine/cysteine dyad are found at the C-terminal ends of strands β3 and β4, respectively. This basic monomeric fold led to the identification of the other clan CD members and the description of a minimal core structural unit, the caspase/haemoglobinase fold (CHF) [23], which is described as consisting of the first four strands of the β-sheet (2_↑_1_↑_3_↑_4_↑_) along with helices α1–α3 (Figure 1A)_._

A highly conserved proteolytic aspartate is found situated between strands β4 and β5 of the caspases. As a result, the original caspase structures were described as having a large (α or p20) and a small (β or p10) subunit, comprising strands 1–4 and 5–6, respectively, linked together by an inter-subunit linker [4] (a cleaved loop region). This description of two individual caspase subunits predated any 3D structural information [24], although the term ‘inter-subunit linker’ was most probably introduced later. In reality, caspase monomers do not contain individual subunits but are simply composed of a single polypeptide chain, which folds into a central six-stranded β-sheet with a highly conserved cleavage site. In addition, because of the abundance of caspase structures available in the literature, other important loop regions have been named in various ways. Therefore, to standardize the nomenclature used in the present review, and to allow structural comparisons with other families in the clan, all terms referring to caspase subunits are omitted and the loop (L) regions are named according to the strands that they follow (L1–L5, respectively) (Figure 1A). Consequently, the substrate-binding loop regions in the caspases that have been historically known as the 179 loop, inter-subunit linker, 341 loop and 381 loop (caspase-1 nomenclature [18]) or L1, L2, L3 and L4 (caspase-7 nomenclature [25]) are simply referred to as loops L1, L4, L5 and L5(L5 after α5), respectively (Figure 1A).

**Figure 2:**
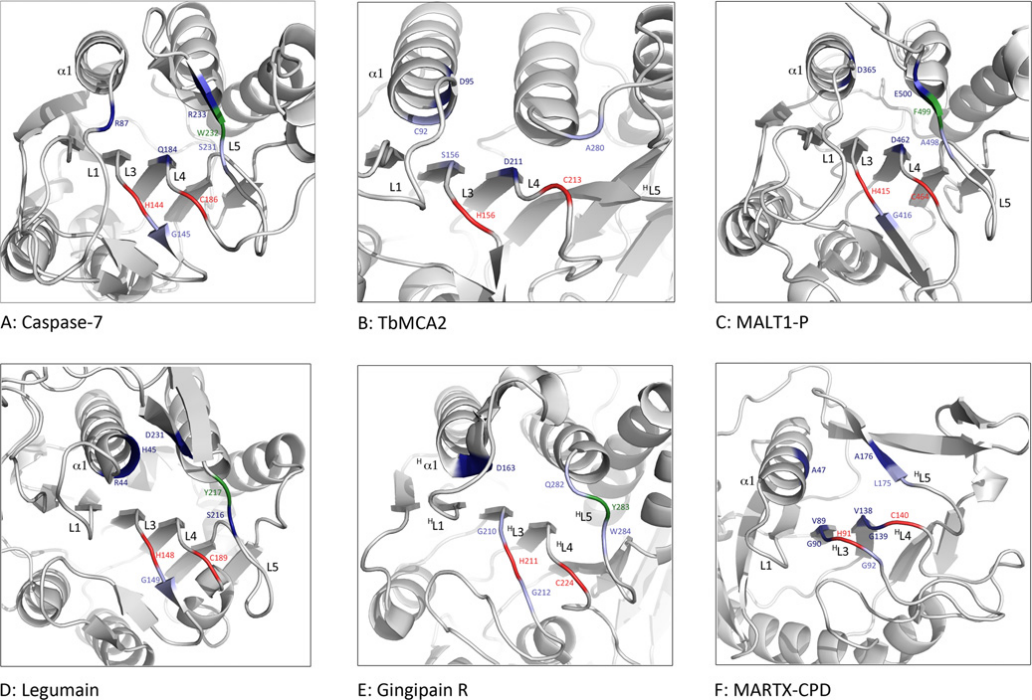
The S_1_-binding pockets of the clan CD family members The catalytic dyad is shown in red and conserved aromatic residues are shown in green. With the exception of TbMCA2, residues that form hydrogen bonds to the P_1_ residue of a bound inhibitor are shown in blue (the darker shade of blue represents interactions through functional groups, whereas the lighter blue shows interactions from main chain atoms). Residues and SSEs involved in P_1_ binding are labelled and SSEs structurally homologous (but topologically diverse) to those found in the caspases are highlighted (^H^). (**A**) Caspase-7 PDB (ID 1F1J). (**B**) Inhibitor-free TbMCA2 (PDB ID 4AFR) in which residues shown to be important in substrate binding are highlighted in blue, with those responsible for specificity in P_1_ [11] shown in navy blue. (**C**) MALT1 paracaspase domain (MALT1-P) (PDB IB 3UOA). (**D**) Legumain (PDB ID 4AW9). (**E**) Gingipain R (PDB ID 1CVR). (**F**) MARTX-CPD (PDB ID 3GCD). Inhibitors used in complex structures are shown in Supplementary Table S1.

Many caspase structures have been determined complexed with an inhibitor bound in the active site, and analysis of such structures allows the hydrogen bond interactions and hydrophobic contacts to the bound inhibitor to be identified (using LigPlot+ [26], see Supplementary Figure S1). Correspondingly, the structure of caspase-7 in complex with the inhibitor acetyl-Asp-Glu-Val-Asp-aldehyde [21] (Ac-DEVD-CHO, see Supplementary Table S1) reveals that there are a total of five residues responsible for hydrogen bonding to the P_1_ aspartate on the inhibitor: Arg^87^, Gly^145^, Gln^184^, Ser^231^ and Arg^233^. Mapping these residues on to the corresponding regions in the structure, using the PyMOL molecular graphics system, version 1.2r3pre (http://pymol.sourceforge.net, Schrodinger) reveals that they are found on loops L1, L3(β^1^), L4, L5 and L5(β^2^), with the catalytic dyad (His^144^/Cys^186^) sitting on L3 and L4, respectively (Figure 2A). Only the functional groups of Arg^87^, Gln^184^ and Arg^233^ interact with the carboxylic acid side chain of the P_1_ aspartate, suggesting that these three residues are collectively responsible for the specificity of the S_1_-binding site. Notably, Arg^233^ (on L5β^2^) forms a small section of β-sheet with the bound inhibitor but, in the absence of inhibitor, this region of active caspase-7 [28] (see Supplementary Table S1) does not contain any discernible secondary structure. In addition, several of the effector caspases can be inhibited by the X-linked inhibitor of apoptosis (XIAP) family of proteins and, in the case of caspase-7, the interactions with XIAP closely resemble those between caspase-7 and Ac-DEVD-CHO (see Supplementary Table S1 [29]).

All known caspases form antiparallel homodimers and, despite the fact that each monomer contains a catalytic dyad and active site, dimerization is critical for the stability and formation of a fully mature caspase. The caspase dimer is formed through β-strand–β-strand interactions along β6, resulting in an extended 12-stranded β-sheet that is very stable under physiological conditions [19,30] (see Figure 1). However, depending on the type of caspase, dimerization occurs at different points in the activation process [19]. In general, the inflammatory and initiator caspases are present in cells as monomers and activated by dimerization, whereas the effector caspases exist in cells as dimeric zymogens and are activated by intermolecular cleavage (often by an initiator caspase) at the conserved aspartate in L4. Activation by cleavage generally results in a large movement in cleaved L4, creating space, which allows the active site loops to adopt the correct orientation for activity. Typically, the cleaved ends of L4 cross the dimer interface and stabilize the substrate-binding groove in the opposite dimer, without contributing directly to the opposing active site. It is interesting that activation of monomeric caspases by dimerization is often followed by a maturation event such as removal of the prodomain or cleavage of the L4 loop [20] and, although these events can contribute to overall stability [31], they are not required for full activation, because a fully functional active site is formed in their absence.

Activation by dimerization of the initiator and inflammatory caspases is facilitated by their extended prodomains via an activation platform, whereby adaptor proteins recognize and bind the N-terminal recruitment domains (reviewed in Pop and Salvesen [20]). However, the role of the shorter prodomains found in the effector caspases is less well defined. Typically, the term ‘prodomain’ refers to a region in an enzyme that must be removed for, or before, activation. However, in caspase-6 the N-terminal region was shown to inhibit autoactivation [32] by preventing intramolecular cleavage on L4 [33], but removal of the region had an adverse effect on stability and no effect on the enzyme's activity against peptide substrates [34]. Similarly, the prodomain of caspase-3 was shown to keep the enzyme in a latent form until its activation by a downstream caspase [35], but removal of the N-terminal region had no effect on the enzyme's activity [36]. This also holds true for caspase-7, for which the catalytic activity of the proform is indistinguishable from that of the wild type [28] and the cleaved form is reported to be more apoptotically active [37]. Consequently, in contrast to some other peptidases (such as the clan CA peptidases, reviewed by Turk et al. [38]), removal of the effector caspase prodomain is not necessary for catalytic activity, although it may play an inhibitory role until enzymatic activity is required. In addition, the N-terminal region of an effector caspase has so far escaped structural elucidation. This is despite the fact that both caspase-6 [33] and caspase-7 [28] (see Supplementary Table S1) have been crystallized as inactive proenzymes, suggesting that the N-terminal regions do not bind (tightly) to the surface of the enzymes and are assumed to be reasonably flexible in solution.

### Metacaspases

Given the importance of the caspases in mammals, a search for orthologues in plants and other non-metazoan organisms was undertaken by using the primary sequences of caspases, in and around the active site, in a PSI-BLAST (Position-Specific Iterative Basic Local Alignment Search Tool) search [39]. This resulted in the identification of two new groups of peptidases that were collectively assigned in MEROPS as a new caspase subfamily (C14B). These peptidases, termed ‘paracaspases’ and ‘metacaspases’, were both found to be present in the genomes of bacteria and Archaea [40,41]. In addition, the metacaspases were identified in protozoa, fungi and plants [39], whereas the paracaspases were found distributed throughout the animal kingdom, from which the metacaspases were notably absent [42] (see Table 1).

Early studies on the metacaspases attempted to draw parallels between possible metacaspase function and the fundamental and well-established processes carried out by the caspases [43–46]. Indeed, the yeast metacaspase Yca1 (from *Saccharomyces cerevisiae*) has been implicated in cell death processes [43], suggesting a degree of functional homology with the caspases. This resulted in similar investigations being carried out on metacaspases from other organisms, and revealed a role for several fungi and plant metacaspases in cell death (reviewed in Tsiatsiani et al. [47]). However, a link with cell death mechanisms could not be identified for all metacaspases and a number of other functions have since been established in various cellular processes including cell-cycle progression [48], cell proliferation [49], endoplasmic reticulum (ER) stress [50], clearance of insoluble aggregates [51] and virulence [52].

Historically, two types of metacaspases have been described [39] (types I and II), with both types being found in plants whereas yeast and the protozoa possess only type I. In addition, a further type of metacaspase (denoted type III) has recently been described in unicellular photosynthetic algae and bacteria [40]. It is of interest that the number and type of metacaspase genes identified in different organisms can vary considerably [47], although there is insufficient evidence to indicate whether this shows a degree of functional specialization or redundancy; both have, however, been reported [44,50,53–56]. In addition, multi-functional metacaspases have also been identified, particularly in organisms that have a single metacaspase gene, e.g. in *S. cerevisiae* Yca1 [43,48,51] and *Leishmania major* LmMCA [57–59].

The original structural classification of all three types of metacaspases is based on a predicted domain structure originating from the system adopted for the caspases. This describes metacaspases as containing large (p20) and small (p10) subunits, with the addition of other variable structural features such as an N-terminal prodomain (type I), an extended inter-subunit linker (type II) and a putative p20/p10 domain swap (type III) [40]. However, in contrast to the caspases, active metacaspases show a strict preference for substrates containing basic arginine and/or lysine residues [46,59–61] (see Table 2). Indeed, this preference for basic substrates makes the name ‘meta**casp**ase’ technically incorrect. Metacaspases also differ significantly from the caspases in that they are active monomers [11], for which activation profiling has revealed a widespread, but not universal [62], requirement for calcium [45,60,63,64]. In addition, there are no conserved cleavage sites reported in type I metacaspases, but this is different for type II metacaspases, which contain highly conserved cleavage sites that have been shown to play an important part in the activation mechanism of *Arabidopsis thaliana*, AtMC4 and AtMC9 [61,65].

Despite being described as having an N-terminal prodomain, there is no evidence in the type I metacaspases for removal of this region for activation. However, several distinct functions have been attributed to the N-terminal regions in type I metacaspases: in AtMC1, the N-terminal domain contains a conserved LSD1-like (lesion-simulating disease 1-like) zinc finger motif, which was found to interact with LSD1 and negatively regulate its function [44]; in Yca1, the N-terminal domain is essential for targeting the enzyme to insoluble aggregates [51] and in *Trypanosoma brucei* MCA2 (TbMCA2) the N-terminal region is thought to act as a gatekeeper, controlling substrate access to the active site [11]. In addition, stronger autoprocessing has been observed in AtMC1 and AtMC2 when the N-terminal region is absent [44], suggesting that, similar to TbMCA2 (and the effector caspases), this region acts to inhibit/control enzymatic activity until it is required.

#### TbMCA2 structure

**Figure 3:**
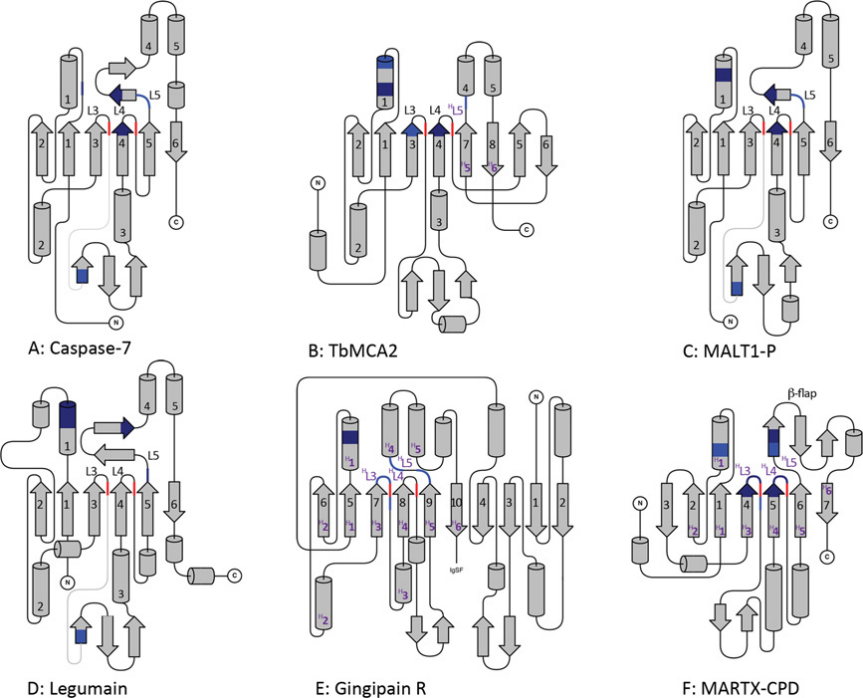
The structural topologies of the clan CD enzymes (**A**) Caspase-7; (**B**) TbMCA2; (**C**) MALT1-P; (**D**); legumain; (**E**) gingipain R; and (**F**) MARTX-CPD. The S_1_-binding pockets are highlighted as in Figure 2 and the topologies are based on the PDB codes described in the same Figure. Strands in the central β-sheet are numbered from the N-terminus in black. Black numbering is also used for the five major α-helices and important S_1_-binding loops (L) when they are located in the structure in the same order as they are in the caspases. SSEs that are structurally homologous to those found in the caspases, but appear in the structure in a different order, are highlighted with an (^H^), followed by the caspase numbering, and shown in purple (α and β have been omitted as a result of space constraints but are used in the text). The position of the catalytic dyad (H/C) is shown in red on loops L3 and L4 (or ^H^L3 and ^H^L4), respectively.

The structural basis for many of the functional differences between the metacaspases and caspases was revealed by the first metacaspase structure, an inactive (C213G/A) mutant of TbMCA2 [11]. The overall topology of this metacaspase was rather unexpected, because the structure did not contain the six-stranded β-sheet found in the caspases. Instead, TbMCA2 contained two extra strands (β7 and β8) sandwiched between β4 and β5, resulting in a monomeric structure with an eight-stranded β-sheet of 2_↑_1_↑_3_↑_4_↑_7_↑_8_↓_5_↑_6_↓_ topology [22]. Similar to the caspases, five α-helices and a small section of β-sheet on L3 were found surrounding the central sheet with various loop regions connecting the secondary structural elements (SSEs) (Figures 3A and 3B). However, unlike the caspases, the N-terminal region was extremely well ordered and the 70-residue region preceding β1 was found to encircle the enzyme and cross over the active site.

For direct comparison of structures from different clan CD families throughout the present review, caspase nomenclature is highlighted for the SSEs and loops of all clan CD structures, when they are structurally conserved and similar to those found in the caspases, e.g. β7 in TbMCA2 is structurally homologous to β5 in the caspases–denoted as ^H^β5 (^H^5; Figure 3B).

Structural determination of a metacaspase in the presence of a bound substrate and/or inhibitor has so far escaped elucidation and as a consequence the metacaspase S_1_-binding pocket cannot be mapped in the same way as the caspases. However, a potential S_1_-binding pocket was described for TbMCA2 and several residues were shown to be involved in substrate binding and/or enzyme activity [11]: Cys^92^, Asp^95^, Ser^156^ and Asp^211^, which were found on α1, α1, L3 and L4, respectively (see Figures 2B and 3B). In addition, the L7 (^H^L5) loop of TbMCA2 was shown to undergo a structural rearrangement at Ala^280^ (Figure 3B), in the presence of Ca^2+^, and is also thought to be important in substrate binding [11]. The structure of TbMCA2 was determined in the presence of samarium, which facilitated the identification of an allosteric Ca^2+^-binding site comprising four aspartate residues (Asp^173^, Asp^189^, Asp^190^ and Asp^220^), which are highly conserved in a primary sequence alignment of both type I and type II metacaspases [11]. The PDB identities for the individual structures are referenced in Supplementary Table S1.

#### Yca1 structure

Elucidation of the structure of TbMCA2 was closely followed by that of the crystal structure of another type I metacaspase from yeast, Yca1 [12]. Comparing Yca1 with TbMCA2 revealed that the two structures are very similar, sharing 82% of their SSEs (PDBeFold [66]), and that the predicted S_1_- and Ca^2+^-binding sites in TbMCA2 are completely conserved in Yca1, in terms of both structure and residue type (see Supplementary Figure S2). Unlike TbMCA2, the structure of Yca1 was determined from full-length active protein (residues 1–432), although the enzyme was treated with V8 peptidase before crystallization [12] and, consequently (or otherwise), the structure of Yca1 contains four regions with missing residues: the N-terminus (89 residues), the turn of the β-hairpin on L3 (11 residues), L6 (51 residues) and L7 (^H^L5) (11 residues). It is of interest that these regions are relatively diverse between TbMCA2 and Yca1, with L6 and the N-terminal region being the most notable (see Supplementary Figure S2). In TbMCA2, L6 is 8 residues long (and well ordered), whereas in Yca1 it is 59 residues long and disordered, making L6 a potentially interesting variation between the two enzymes. In addition, compared with TbMCA2, Yca1 has an extended, non-conserved, N-terminal region (136 as opposed to 70 residues, respectively). The first 68 residues of Yca1 consist of QXXQ repeats involved in targeting Yca1 to insoluble aggregates in yeast [51] and, although the first 89 residues are absent in the structure, a further 48 N-terminal residues are found to be ordered. However, unlike TbMCA2 these residues do not wrap around the enzyme, but rather are found cradling the base of β5–β8 with a small β-hairpin section running parallel to the missing region in L6.

### Paracaspases

Paracaspases are the second group of enzymes classified in the caspase subfamily C14B and, similar to the metacaspases, these enzymes recognize basic substrates, cleaving after arginine residues (see Table 2). To date, the only available paracaspase structures come from the human and murine mucosa-associated lymphoid tissue translocation protein 1 (MALT1) [10,67]. MALT1 is a large multi-domain protein, which exhibits functionally important, arginine-specific, proteolytic activity as a result of its paracaspase domain. The full-length protein comprises an N-terminal death domain (DD), followed by two immunoglobin (Ig)-like domains (Ig1 and Ig2), the paracaspase domain, a further Ig-like domain (Ig3) and approximately 100 C-terminal residues with no apparent secondary structure [10]. The recombinant peptidase appears to be more stable (remains soluble in solution) *in vitro* when it is expressed as a complex of the paracaspase/Ig3 domains [67,68], although the paracaspase domain alone is active [68].

The original crystal structures of MALT1 were obtained for the apo-catalytic domain and the paracaspase/Ig3 domains with and without the peptide inhibitor benzoxycarbonyl-Val-Arg-Pro-Arg-fluoromethylketone (Z-VRPR-FMK) [10,67] (see Supplementary Table S1).

The structure of the MALT1 paracaspase domain (MALT1-P) has a fold virtually identical to that of all known caspases [10] (see Figure 3C). In addition, MALT1 requires dimerization to gain activity [67] and the structures both revealed an antiparallel caspase-like dimer along β6. However, unlike the caspases, cleavage in L4 is not required for activation and/or maturation of the enzyme and this is obvious from the inhibitor-bound form of the structure, which shows L4 to be intact and well ordered. Conversely, L4 is disordered in the apo-structure, suggesting that the inhibitor and/or a substrate is required to stabilize this loop. The structure of MALT1-P with Z-VRPR-FMK reveals that four residues are involved in hydrogen bonding to the P_1_ arginine of the inhibitor: Asp^365^ (α1), Gly^416^ (L3), Ala^498^ (L5) and Glu^500^ (L5). In addition, Asp^462^ (L4) is also found in the S_1_-binding pocket, suggesting that Asp^365^, Glu^500^ and Asp^462^ are responsible for the substrate specificity of MALT1 in P_1_ (see Figure 2C).

Apart from the ordering of L4, the most striking difference in the apo- and inhibitor-bound forms of MALT1 is found within L5, which undergoes a significant structural rearrangement, repositioning an important glutamine residue. In the ligand-free structure, this residue (Gln^494^) points directly into the S_1_-binding pocket, blocking access to the active site. However, in the inhibitor-bound form L5 points away from the main body of the enzyme, towards the solvent, and forms an elbow with Gln^494^ sitting at the tip [69]. This is a substantial shift in Gln4^94^ between the two structures of approximately 13 Å (1 Å=0.1 nm) and approximately 180°; when the inhibitor is bound L5 forms a small β-strand–β-strand interaction with the inhibitor, as observed in caspase-7. The conformation of Ig3 also changes on inhibitor binding, leading to the suggestion that MALT1 activation is a two-step process relying on both dimerization and, on substrate binding, release from Ig3-mediated autoinhibition [69].

### Comparison of family C14

As described above, there are two diverse substrate specificities exhibited within family C14, with the caspases (C14A) recognizing acidic aspartate residues whereas both the metacaspases and the paracaspases (C14B) recognize basic arginine and/or lysine residues (see Table 2). Despite this, the structure of MALT1-P has much more similarity with the caspases than the metacaspases. Indeed, the overall topology of paracaspases and caspases, with six-stranded β-sheets, is virtually identical [10], forming structurally homologous active dimers (see Figures 3A and 3C).

To investigate the structural similarities, 3D pair-wise structural alignments of caspase-7 with MALT1-P and TbMCA2, along with the alignment of MALT1-P with TbMCA2, were carried out using PDBeFold [66] (Table 3). This reveals that 79% of the SSEs in MALT1-P can be identified in caspase-7. In addition, the two enzymes align with 19% sequence identity: a root-mean-square deviation (RMSD) of 1.94 on the Cα positions over 177 aligned residues and a *Q* score [66] of 0.41. Here, the *Q* score represents the quality of the Cα alignment by taking into account both the RMSD and the alignment length (where a protein matched with itself will have a *Q* score of 1) (Table 3).

**Table 3:** Three-dimensional superposition of clan CD families with caspase 7 The Table is ordered in terms of the quality of the Cα alignment (Q score, Q^S^), in which %SSE^Q-C7^ is the percentage of the SSEs in the query (*Q*) that can be identified in caspase-7 (where *Q*=MALT1-P, legumain, TbMCA2, PmC11, gingipain R and MARTX-CPD); %SSE^C7-Q^ is the percentage of the SSEs in caspase-7 that can be identified in *Q* (see above); % Seq. ID is the percentage of the sequence identity found after structural alignment; *N*_align_ is the number of matched residues; and RMSD is the root-mean-square deviation on the Cα positions of the matched residues.

Enzyme	Family	PDB ID	*Q*^SH^	%SSE^Q-C7^	%SSE^C7-Q^	% Seq. ID	*N*_align_	RMSD (Å)
Caspase-7	C14A	1F1J	1.00	100	100	100	230	0.00
MALT1-P	C14B(P)	3V4O	0.41	79	73	19	177	1.94
Legumain	C13	4AW9	0.34	65	87	13	173	2.05
TbMCA2	C14B(M)	4AFR	0.22	59	67	13	175	2.69
PmC11	C11	3UWS	0.14	38	73	11	151	3.03
Gingipain R	C25	1CVR	0.13	32	67	9	161	2.97
MARTX-CPD	C60	3GCD	0.10	47	47	6	109	3.60

Superimposing TbMCA2 over caspase-7 revealed that the two structures align over a similar number of residues to MALT1-P and caspase-7 (175 with an RMSD of 2.69). However, this alignment also confirmed that TbMCA2 is much less similar to the caspases than MALT1-P, for which the SSEs in caspase-7 account for only 59% of the SSEs in TbMCA2, with a much lower *Q* score (0.22) and a sequence identity of only 13% (Table 3). The difference in the metacaspase structure results from the insertion of the two strands, β7 and β8, which form the extended eight-stranded β-sheet. This extended β-sheet appears to stabilize the type I metacaspases as functional monomers, making it impossible for them to use the same dimerization interface as the caspases and paracaspases and, in fact, strands β5 and β6 in the metacaspases are structurally homologous to β5′ and β6′ from the antiparallel dimer in the paracaspases and caspases (see Figures 1 and 3). In addition, the metacaspase structures revealed reasonably large and well-ordered N-terminal regions, which have shown good association with the main body of the enzyme [11]. This differs from the caspases, for which the N-terminal region has never been observed in a crystal structure, suggesting that it is more easily dissociated.

Both type I metacaspases and the paracaspases differ from the caspases in that they do not require or exhibit processing for activity or maturation. However, several type II metacaspases contain a highly conserved basic (arginine or lysine) residue, which is critical for activation [61,65]. This cleavage site is found just before β5 in TbMCA2 at the end of a type II-specific insertion of approximately 70 residues (after L4). It is also not structurally homologous to the processing site in the caspases, which occurs at about 14 residues after β4, as opposed to about 84 residues in type II metacaspases. This suggests that the mechanism for type II metacaspases is structurally distinct from both type I metacaspases and caspases.

Comparing the identified active site regions in caspase-7, MALT1 and TbMCA2 reveals that, despite the differences in structure and substrate specificity, they all use structurally homologous regions for inhibitor/substrate recognition, and that the catalytic histidine/cysteine dyad resides in the same positions on the L3 and L4 loops, respectively (see Figure 2). Accordingly, the conserved regions responsible for inhibitor binding in family C14 are L1 or α1 (similar spatial region), L3, L4 and L5 (excluding TbMCA2). In the metacaspases ^H^L5 has not been identified as contributing to the active site because it is disordered in both crystal structures. However, a conformational change was observed in the ^H^L5 loop of TbMCA2 when it was crystallized in the presence of Ca^2+^ [11]. Of interest, this loop is also disordered in both pro-caspase 7 [28] and ligand-free MALT1 [67], but is well defined in the inhibitor-bound enzymes, suggesting that ^H^L5 will also be important in the recognition and/or binding of metacaspase substrates.

The ligand-free structures of both MALT1-P and TbMCA2 revealed that the active sites were blocked sterically, and so most probably autoinhibited, by a residue on a loop region in the structures (Gln^494^ on L5 in MALT1-P and Tyr^31^ on the N-terminal region of TbMCA2). In addition, the Ca^2+^-induced conformational change in TbMCA2 at ^H^L5 resulted in this loop forming a small section of β-sheet with the N-terminus as it crossed the active site. This is similar to the β-strand–β-strand interactions observed between L5 and bound inhibitors in both the caspases and MALT1, suggesting that this Ca^2+^-induced loop movement in TbMCA2 could mimic the conformational change required by ^H^L5 to bind to a peptide/protein substrate.

This structural family of enzymes, classed as C14, collectively exhibits a variety of substrate specificities, activation mechanisms, potential autoinhibitory machinery and N-terminal functionality. Structurally, the specificity-diverse caspases and paracaspases are almost identical whereas the metacaspases have a different structural topology, and all the family members appear to use analogous structural elements to recognize and bind their substrates. Regardless of the diversity exhibited by the family, it is fair to say that the monomeric forms of these structures (caspases, paracaspases and type I metacaspases) are all single-subunit, single-domain monomers, which, in the case of the caspases and paracaspases, form homodimers. Correspondingly, the widespread nomenclature that describes this family as containing a homodimer of heterodimers [3,4], and/or consisting of small and large subunits [18], may need to be reconsidered. In addition, this analysis suggests that the metacaspases are sufficiently structurally and functionally diverse to be classed separately from the caspases and paracaspases; to investigate this fully, however, the structure–function relationships for other available clan CD family members need to be considered.

## FAMILY C11: CLOSTRIPAIN

The archetypal member of family C11 is clostripain: a cysteine peptidase released by the anaerobic bacterium *Clostridium histolyticum.* This family of petidases is reportedly found in most phylogenetic kingdoms but is missing from the Metazoa (see Table 1). Clostripain is reportedly arginine specific, requiring Ca^2+^ for activity and/or stabilization [70]; it needs the loss of an N-terminal pro-peptide, along with cleavage and removal of an internal nine-residue peptide, for full activation [71]. To date, there are no structures of clostripain available in the PDB, but there is a structure of an unassigned peptidase from family C11. The Joint Centre for Structural Genomics [72] determined this structure from the bacterium *Parabacteroides merdae*, under the gene name PARMER_00083 (PmC11, see Supplementary Table S1). The primary sequence of PmC11 is almost 150 residues shorter than that of clostripain but the two enzymes share a primary sequence identity of 23% (Clustal Omega [73]).

**Figure 4:**
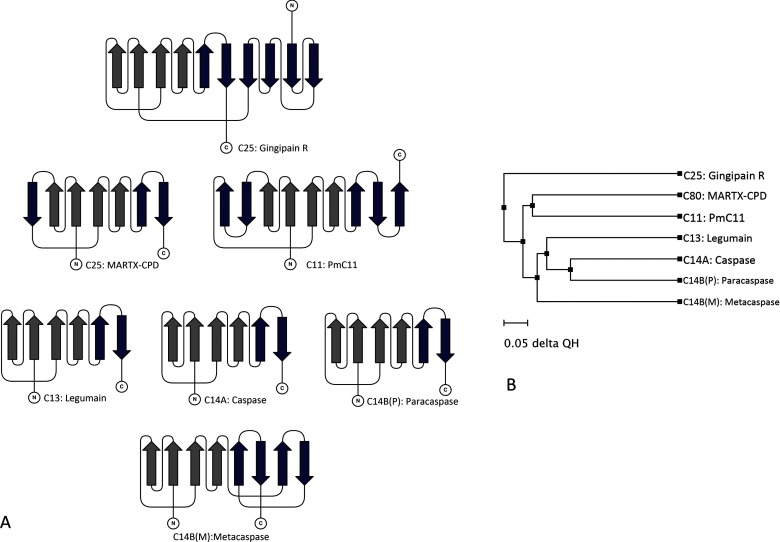
The structural diversity in the central β-sheet of the clan CD enzymes (**A**) The β-sheet topologies exhibited by the families in the clan. The β-strands described by the CHF [23] are shown in grey whereas the other strands are shown in blue; the N- and C-terminal ends of the enzymes are labelled accordingly. (**B**) A phylogenetic tree based on structure, in which *Q*^H^ [85] is a measure of structural homology. This Figure was produced using a STAMP [87] structural alignment and VMD [87].

The structure of PmC11 has a nine-stranded β-sheet with 4_↑_3_↓_2_↑_1_↑_5_↑_6_↑_7_↓_8_↓_9_↑_ topology, in which β1–β2 and β5–β8 overlie well with the six-stranded β-sheet exhibited by the caspases (Figure 4). Correspondingly, the His^133^ and Cys^179^ residues found at the ends of strands β5 and β6 (^H^β3 and ^H^β4, respectively) are likely to be the catalytic dyad. PmC11 also contains five α-helices, which are structurally homologous to α1–α5 in the caspases. Apart from its extended β-sheet, PmC11 differs most significantly from the caspases at its C-terminus, where a further seven α-helices and two β-turns are located after β8 (^H^β6).

## FAMILY C13: LEGUMAIN

The archetypal member of family C13 is legumain, an asparagine-specific cysteine peptidase, which is found throughout most phylogenetic kingdoms (see Table 1), although it has been most extensively studied in the blood fluke parasite *Schistosoma* sp., mammals and plants (in which it was originally identified [74]). Legumain is synthesized as an inactive zymogen with the first 17 residues consisting of a signal peptide, which is released during secretion. Historically, the remainder of the enzyme has been described as consisting of an eight-residue, N-terminal pro-peptide, a peptidase domain and a large 110-residue, C-terminal prodomain. However, the N-terminal region of legumain has recently been shown to have no role in the activation of the enzyme [75], whereas the C-terminal domain has been shown to be instrumental in controlling the zymogen, along with enzyme activation and stability [9].

Currently, the only structures available from family C13 are those recently determined for human legumain [9], including the structure of pro-legumain (peptidase- and C-terminal domains), along with three structures of the peptidase domain complexed with the tetrapeptide inhibitor Ac-YVAD-CMK, at pH 7.5 and pH 5.0, and complexed with the peptidomimetic inhibitor Z-Ala-Ala-AzaAsn-CMK [9] (see Supplementary Table S1). It is interesting that the peptidase domain of legumain is topologically equivalent to the caspases and paracaspases, with a central six-stranded β-sheet and five major α-helices (see Figure 3D). However, unlike the structurally similar C14 enzymes, legumain is active as a monomer despite no initially obvious structural reason for prohibiting caspase-like β6–β6′ dimerization.

Legumain is also distinct from all the C14 enzymes, in that it is activated by pH. Lowering the pH to <5.5 activates the enzyme, with full peptidase activity at around pH 4.0. This acidification is accompanied by intermolecular (*trans*) autoproteolytic processing at Asn^323^, a cleavage site situated in the C-terminal domain. Cleavage is not required for activity but the reaction rate is much faster when cleavage occurs [76]. In general, legumain exhibits specificity towards asparagine in P_1_ (pH optimum 5.5), but at pH ≤4.5 aspartate is accepted (and this specificity becomes a preference for aspartate at about pH 4.0). The functional groups of Arg^44^ (α1), His^45^ (α1), Ser^216^ (L5) and Asp^231^ (L5) contribute to this specificity, producing a zwitterionic S_1_-binding pocket (see Figure 2D), the geometry of which suggests that Asp^231^ is protonated at low pH in order to accept an aspartate in P_1_ [9].

The structure of pro-legumain revealed that the C-terminal domain (C domain) is organized into two distinct parts: an activation peptide (AP, positioned immediately after the peptidase domain) and a C-terminal DD-like fold, consisting of five α-helices, denoted as LSAM (legumain stabilization and activity modulation) domain [9]. Both the AP and the LSAM domains interact extensively with the peptidase domain at the autoprocessing site Asn^323^ found at the interface between them. In addition, Ser^307^ from the AP forms hydrogen bonds to Arg^44^ and Ser^216^ in the P_1_ pocket, blocking access to the active site.

The interacting surfaces of the C domain and the peptidase domain are complementary (positively and negatively charged, respectively) and, as the pH is lowered and the peptidase becomes protonated, the interaction between the two surfaces is disrupted (in particular several salt bridges), which produces a conformational arrangement that allows substrates to access the active site. However, the C domain does not dissociate from the enzyme on processing; in fact it becomes markedly unstable if the LSAM domain is removed [9] and it is not possible to express legumain in the absence of the C domain [75]. Furthermore, superimposing a copy of pro-legumain on to each monomer in the caspase-7 dimer reveals a steric clash between LSAM domains. This, together with the fact that there is no biological need for dimerization, suggests that monomeric legumain is a more energetically favourable form. In summary, a change in pH (the activation mechanism), followed by *trans*-autoprocessing and a conformational rearrangement, has a role in producing fully mature legumain.

## FAMILY C25: GINGIPAIN

The only structure available from the C25 family of clan CD peptidases is gingipain R (RgpB), a virulence factor participating in the infection and survival of *Porphyromonas gingivalis* in periodontitis. To date, the RgpB structure has been determined in both its mature and its pro-forms [13,14] (see Supplementary Table S1). The crystal structure of the mature form of RgpB revealed a monomeric enzyme with a central 10-stranded β-sheet, which is the largest central β-sheet of all the clan CD structures determined to date [13]. Similar to other clan CD structures, the central sheet is surrounded on both sides by β-hairpins and several α-helices. Consideration of the structure in the same orientation as the caspases reveals that the sheet exhibits 6_↑_5_↑_7_↑_8_↑_9_↑_10_↓_4_↓_3_↓_1_↓_2_↓_ topology, resulting in an internal quasi-symmetry situated between strands 9 and 10. However, the four N-terminal strands (β1–β4) are twisted out of the plane of the other strands by about 45°, and are often, perhaps best described as a separate N-terminal subdomain (NSD) [13,14]. The C-terminal subdomain (CSD [14]), encompassing strands β5–β10, overlies well with the structure of caspase-7, including the position of the caspase helices α1–α4 (helices ^H^1–^H^4, see Figure 3). The SSEs in the CSD are named in line with the caspase nomenclature (as described for the metacaspases above), e.g. RgpB β5 is described as ^H^β1 (strand ^H^1, see Figure 3E). The last 84 C-terminal residues after β10 (^H^β6) form an IgSF (Ig superfamily) domain.

RgpB exhibits an exclusive specificity for arginine in P_1_ and the original structure of RgpB was reported complexed with the peptide inhibitor D-FFR-CMK [13]. Analysis of the active site revealed that, similar to other members of clan CD, the catalytic histidine/cysteine dyad (His^221^/Cys^244^) in RgpB is found at the C-terminal ends of ^H^β3 and ^H^β4, respectively. In addition, residues forming hydrogen bonds to the P_1_ arginine are Asp^163^ (^H^α1), Gly^210^ (L3), Gly^212^ (L3), Gln^282^ (^H^L5), and Trp^284^ (^H^L5) (Figure 2E). Trp^284^ is also found stacking on top of the P_1_ arginine covering the S_1_ pocket like a lid. It is interesting that the only functional group involved in hydrogen bonding to the P_1_ arginine is the carboxylic acid of Asp^163^, which forms a stable bidentate salt bridge with the guanidino group.

The pro-form of RgpB consists of a 229-residue, N-terminal prodomain, which contains two autoprocessing sites at Arg^126^ and Arg^229^ (proform numbering denoted ^P^Arg^126^ and ^P^Arg^229^, respectively). Cleavage at these sites is required for full activation of the enzyme [77], with cleavage at ^P^Arg^126^ being essential for processing at ^P^Arg^229^ and subsequent removal of the prodomain. Despite this requirement for full activation, a ^P^R126A/^P^R229A mutant enzyme was found to exhibit some latent activity, albeit 80-fold lower than that of the mature enzyme [77]. Recently, the crystal structure of the inactive RgpB complexed with its prodomain was determined (see Supplementary Table S1), revealing the largest structurally classified prodomain in clan CD to date [14].

This structure revealed that a loop on the prodomain (termed the ‘inhibitory loop’) runs towards the S_1_-binding pocket, injects ^P^Arg^126^ into the pocket, and loops back at this point. This mimics the P_1_ arginine of a bound substrate, with the guanidino group overlying in an identical fashion to that of the bound inhibitor. Similar to the P_1_ arginine of the bound inhibitor, ^P^Arg^126^ makes a bidentate salt bridge with Asp^163^ in the mature enzyme but in addition it makes a strong hydrogen bond with His^221^. This appears to cause ^P^Arg^126^ to rotate away from the catalytic cysteine, with the resulting position being unfavourable for cleavage to occur. However, it is at this position that the initial cleavage for complete activation of RgpB takes place. Of interest, there are very few changes between the structures of the inhibited and the prodomain complexes, suggesting that the zymogen is in a favourable conformation for activity and inhibition by the prodomain is most probably mediated by a competing substrate [14]. However, the mechanism for prodomain dissociation *in vivo* is still unknown.

It is well documented that Rgps are stabilized by calcium and inhibited by EDTA [78] and superimposing the prodomain and inhibitor-bound structures reveals three distinct Ca^2+^ binding sites, which are highly conserved between the two structures. Two of these are found near important substrate-binding regions (^H^L4 and beneath the S_1_-binding pocket). The importance of the third site is less obvious but it does involve Asp^103^ and Glu^107^, which form hydrogen bonds with the prodomain in the structure of the zymogen. It has not been reported whether any of these sites is critical in the activation of the enzyme, but they do all appear to have a role in the structural stability of RgpB with and without the prodomain.

## FAMILY C80: MARTX-CPD

The first structure available for family C80 was that of the CPD from the multi-functional autoprocessing repeats in toxin (MARTX) toxin from the pathogenic bacterium *Vibrio cholerae*. MARTX is an unusually large toxin (>450 kDa), which is secreted by the bacterium, causing disassembly of the actin cytoskeleton, and subsequent bacterial colonization, of the small intestine [79]. The N- and C-terminal regions of the toxin have large sections of conserved repeats with only the central region (about 1700 residues) containing the effector domains which are thought to impart distinct functions to the toxin. One such domain is the CPD, activity of which is required for activation of the toxin in eukaryotic cells [80], via CPD-mediated proteolysis of regions between the various effector domains, to release them [81]. MARTX-CPD itself is activated by *myo*-inositol hexakisphosphate (Ins*P*_6_), a molecule present in the cytosol of eukaryotic cells but absent in the bacterium, hence the activation and subsequent processing of the toxin would not occur until after translocation into the host.

In addition to *V. cholerae* MARTX toxin, the CDP domains of *Clostridium difficile* toxins A and B (TcdA and TcdB, respectively) have also been determined [16,17] (see Supplementary Table S1). These enzymes are also activated by Ins*P*_6_ and similarly require CDP-mediated autoprocessing to allow for overall toxin function [82,83]. The structure of MARTX-CPD contains a seven-stranded β-sheet with 3_↓_2_↑_1_↑_4_↑_5_↑_6_↓_7_↓_ topology, which is surrounded by three major α-helices (see Figure 3F). The structures of TcdA-CPD and TcdB-CPD are topologically identical to each other and very similar to the structure of MARTX-CPD, which shares 93% of its SSEs with TcdA-CPD (PDBefold [66]). However, the structures of the *C. difficile* CPDs contain a nine-stranded β-sheet with an extra strand flanking both ends of the MARTX-CPD sheet, giving an overall topology of 4_↑_3_↓_2_↑_1_↑_5_↑_6_↑_7_↓_8_↓_9_↑_; this is the same as observed for PmC11 (see Figure 4).

The structure of MARTX-CPD has been determined in its an apo- [15], inhibitor-bound [81] and pre-processed forms [84] (see Supplementary Table S1)–and together these structures provide a fascinating insight into the unusual activation mechanism of the peptidase. Consequently, this enzyme is used for further structural analysis. In MARTX-CPD, strands β1–β2 and β4–β7 overlie reasonably well with the six-stranded β-sheet found in the caspases, with β3 sitting on the outside of this core structure (see Figure 3F). In addition, a β-hairpin loop (βA–βC), termed the ‘β-flap’ [15], is located between ^H^β5 and ^H^β6. Spatially, this replaces α4 and α5 in the caspases and other clan CD members (see Figure 3) and forms a cleft in which Ins*P*_6_ is found.

MARTX-CPD and TcdA/B-CPDs all have a strict specificity for leucine in P_1_ [81], and consequently family C80 is the only family in clan CD, reported to date, that has a preference for a hydrophobic substrate (see Table 2). The first step in the CPD-mediated proteolytic activation of the MARTX toxin [15] is intramolecular autoprocessing of the CPD itself. This internal cleavage occurs at a leucine residue found 30 residues N-terminal to β1 (Leu^0^-Ala^1^ [15], P_1_–P_1_) and occurs only after activation of MARTX-CPD by Ins*P*_6_. In the structure of the inactive and unprocessed form of MARTX-CPD, Leu^0^ is found anchored in the large hydrophobic S_1_-binding pocket, occupying the same position as the P_1_ leucine in the inhibitor (Z-LLL-EP-COO-Et)-bound structure [81] (see Supplementary Table S1).

The catalytic dyad (His^91^ and Cys^140^) is found at the C-terminal ends of ^H^β3 and ^H^β4, and a total of seven hydrophobic residues lie within van der Waals’ bond distance (4.4 Å) of the P_1_ leucine [81]: Ala^47^ (α1); Gly^89^–Val^90^ (^H^L3); Val^138^–Gly^139^ (^H^L4); and Leu^175^–Ala^176^ (βA, ^H^L5β1) (see Figure 2F). In all crystal structures of MARTX-CPD an Ins*P*_6_ molecule is found bound by 13 residues, in a large basic pocket structurally segregated from the S_1_-binding pocket. Notably, both the S_1_- and Ins*P*_6_-binding pockets contain residues from distinct regions on the β-flap, with βA and βB contacting the P_1_ leucine and Ins*P*_6_, respectively.

It has been shown, in the pre-processed form of MARTX-CPD, that Leu^0^ occupies the S_1_-binding site (presumably controlling the latent activity) irrespective of Ins*P*_6_ binding, but not in a way amenable to intramolecular processing; for this Ins*P*_6_ is required [84]. Binding of Ins*P*_6_ causes a movement in βB, which is communicated to the hydrophobic S_1_ residues on βA (via the β-flap), resulting in Leu^0^ becoming more tightly bound in the active site [84]. The outcome of this is that the orientation of the scissile bond in Leu^0^, relative to the catalytic cysteine, becomes amenable to *cis-*cleavage. After Ins*P*_6_-activated processing, the CPD loses its high affinity for the Ins*P*_6_ molecule and is thought to release it. Reoccupation of the S_1_ site by a new substrate is thought to reactivate the InsP_6_-binding affinity and hence the proteolytic activity of MARTX-CPD.

## CLAN CD COMPARISON

### Overview

The caspases are the original structural family in clan CD and, despite growth in structural knowledge for other clan CD families, they remain a sensible structural archetype. This is supported by the fact that many of the SSEs found in the caspases are present in other family members. Indeed, all members of clan CD contain the six-stranded β-sheet exhibited by the caspases [with five parallel and one antiparallel strand(s)], with the catalytic histidine/cysteine dyad found at the C-terminal ends of ^H^β3 and ^H^β4, respectively (see Figures 2 and 3). In addition, all members, with the exception of MARTX-CPD (and the other C80 family members), share five structurally conserved α-helices (^H^α1–^H^α5) (see Figure 3). This is emphasized by aligning the structures from all the other clan CD families with the caspases (using caspase-7 as a template) and obtaining a measure of structural similarity (PDBeFold [66], as described above).

In terms of similarity to the caspases, the *Q* score reveals that MALT1-P is more similar than (>) legumain > TbMCA2 > PmC11 > gingipain > MARTX-CPD. The alignment also shows that 67–87% of the SSEs of caspase-7 can be identified in all other family members, apart from those found in family C80, which shares only 47% of its SSEs with caspase-7, and vice versa (see Table 3). It is interesting to note that caspase-7 shares more of its SSEs (87%) with legumain than with any other family member, making the caspases significantly more structurally similar to legumain than to the metacaspases. This analysis also revealed that MALT1-P (C14B) is more structurally similar to caspase-7 (C14A) than any of the other structures in the clan (see Table 3). The structural differences of legumain, MALT1-P and the caspases are rather subtle, whereas the other families, with much longer central β-sheets, share far fewer of their SSEs with the caspases (from 47% to 32%) (see Table 3). Indeed, the basic topology of the central β-sheets differs in all the families apart from those represented by the caspases, paracaspases and legumain (see Figure 4).

Comparing all the structural families with each other reveals that more of the SSEs in PmC11, legumain, RgpB and MARTX-CPD are found in the caspases than in any of the other structures (see Supplementary Table S2), i.e. none of these families shares more SSEs with each other than with the caspases. However, more of the SSEs in TbMCA2 can be identified in MALT1-P and legumain than caspases, whereas MALT1-P shares the same number of SSEs with both caspase-7 and legumain (see Supplementary Table S2a). Comparison of just the *Q* scores reveals that no two families are more structurally similar to each other than to the caspases, confirming that at a basic level all these structures share basic structural elements with the caspases (see Supplementary Table S2b). However, this scoring method considers only the parts of the structures that align and does not take into account gaps in the alignment, which would account for potentially important deletions and insertions.

Using an adaptation of the *Q* score, known as *Q*^H^ (a measure of structural homology) [85], that takes into account these alignment gaps, a more accurate representation of structural homology can be calculated. To calculate the *Q*^H^ scores, a STAMP [86] multiple structural alignment was carried out on all the families represented by clan CD, using the molecular visualization program VMD [87]. The differences in *Q*^H^ values were derived from the structural alignment and a phylogenetic tree based on these values was produced to depict a structural evolution of the clan (see Figure 4). This phylogenetic tree (based on structure) suggests that legumain, the caspases, the paracaspases and the metacaspases are all more closely related in structure to each other than to the other families in the clan. However, although the caspases and paracaspases are found on the same branch of the tree, both legumain and the metacaspases are found on different, distinct branches. This tree also suggests that PmC11 and MARTX-CDP sit on the same branch, distinct from the caspase group, and that RgpB represents the most structurally distinct family.

### S_1_-binding sites

In general, the families found in clan CD exhibit individual and rather strict substrate specificities, with a preference for basic residues in P_1_ being the most common (see Table 2). Five of the families in clan CD have been determined as complexes with peptide inhibitors–no complex structures are currently available for the metacaspases or PmC11. Mapping the residues involved in substrate binding on to the structures allowed the residues and SSEs in the S_1_-binding pockets to be determined and compared.

In the caspases, substrate recognition has been well studied and is known to depend on three highly conserved side chains from Arg^87^, Gln^184^ and Arg^233^ (caspase-7 nomenclature) that are responsible for creating a basic environment for binding an aspartate residue on P_1_. These residues are found on loops L1, L4 and L5 (the last of which forms a short section of β-sheet with the bound inhibitor), respectively. In MALT1, acidic aspartate and glutamate residues [Asp^365^ (α1), Asp^462^ (L4) and Glu^500^ (L5)] are found in similar structural positions, respectively (see Figures 2A and 2B). Although a functionally specific Glu^500^ in MALT1 is found to overlie with Arg^233^ in caspase-7 (opposite charges, opposite binding specificities), no such charged residues are found in this position in the topologically equivalent legumain. It is interesting, however, that, although legumain does not appear to have a functional substrate-binding residue on L5, the guanidino group from Arg^44^ on α1 overlies in almost exactly the same way in Arg^233^, despite the fact that the Cα positions are found on markedly different parts of the structures. Notably, for all the structures analysed, S_1_ specificity in clan CD can be attributed solely to three main structural regions–^(H)^α1 [^(H)^L1], ^(H)^L4 and ^(H)^L5–and these all appear to contribute to a correctly charged P_1_-binding environment. Analysis of the S_1_-binding pockets of the various families also reveals a conserved aromatic residue on ^(H)^L5 (see Figure 2), which forms hydrophobic contacts with the bound inhibitors (see Supplementary Figure S1) and may be important for directing substrates into the S_1_ pocket.

By looking at the structural regions forming hydrogen bonds and hydrophobic contacts to the inhibitors in the P_2_–P_4_ positions (in addition to P_1_), it becomes obvious that: the regions involved in the S_1_- to S_4_-binding pockets in clan CD are ^(H)^L1, ^(H)^α1, ^(H)^L3, ^(H)^L4, ^(H)^L5, ^(H)^α4 and ^(H)^L6; and, along with the histidine/cysteine dyad, a glycine residue adjacent to the catalytic histidine is also structurally conserved in all families. Mapping of these regions may allow the substrate-binding residues in other, ligand-free, structures to be predicted. Indeed, mapping of the residues known to be important for activity in TbMCA2 on to the SSEs in the structure reveals that these residues are found on α1, L3 and L4. However, ^(H)^L5, which is important for binding in all the complex structures, is disordered in TbMCA2, although it exhibits a shift in the presence of Ca^2+^ [11], suggesting that it will, most probably, be involved in substrate binding in the metacaspases. MARTX-CPD is somewhat different to the other family members and its substrate-binding regions are similar but not quite as conserved as those of the other families. However, even when the SSE in MARTX-CPD has changed from an α-helix to a β-strand (in the case of βA and ^H^α4), the interacting residues in the different families are spatially equivalent. Subsequently, all ligand-binding regions in clan CD are found in parts of the structure that overlie well with the caspases, in front or on top of the β-sheet, as shown in Figures 2 and 3.

### Pro-forms

The structures of the clan CD enzymes reveal that they often contain diverse N-terminal and/or C-terminal (prodomain) regions. With the exception of PmC11, data are available for the N-terminal regions for all the families. In the case of the initiator and inflammatory caspases, the large N-terminal regions contain important CARD or DED domains, whereas the effector caspases have short N-terminal regions for which the function is less well defined. It is difficult to ascertain whether the N-terminal regions in the effectors are true prodomains (as they are often described) because emerging research is starting to suggest that their removal is not necessary for activation; in contrast, however, they do appear to have a part to play in enzyme inhibition. In addition, these regions have eluded structural determination, suggesting that they are not strongly bound to the surface of the caspase. This is rather different to the N-terminal regions found in TbMCA2, RgpB and MARTX-CPD, in which structures determined with the N-terminal regions present revealed that they formed a considerable number of hydrogen bonds on the surface of the peptidase domain.

In addition to binding to the surface of the protease, the N-terminal region in TbMCA2 was found to obstruct the active site by forming hydrogen bonds with residues in the S_1_-binding pocket. Similarly, the N-terminal domain of RgpB injects a residue into the active site, forming a salt bridge with the residue responsible for substrate specificity in P_1_, and in the MARTX-CPD the N-terminal region is found to hydrogen bond to the catalytic cysteine. It is also of interest that, although MALT1 is a large complex with a DD and two Ig-like domains sitting N-terminal to the paracaspase, the apo-form of the enzyme also exhibits an active site obstruction from a residue situated on one of the substrate-binding loops. In addition, although the structure of the N-terminal region of legumain (about 25 residues) is not available, an ordered C-terminal domain is present in the structure of the zymogen [9], which has extensive interactions with the peptidase domain (PDBePisa [88]) and also blocks the S_1_-binding pocket.

This suggests that all these families exhibit some level of proteolytic inhibition until they need to function as active peptidases. In the case of TbMCA2, MALT1 and MARTX-CPD, it is known that ‘inhibitory’ sections of the enzymes do not dissociate from the peptidase on activation, but an obvious movement in these sections is required before substrate binding. It is possible that there is a similar mechanism in some of the effector caspases. Unusually for the clan, the N-terminal domain of RgpB dissociates from the main body of the enzyme and, although the mechanism for this is not entirely understood, it is most probably due to a competing substrate in the presence of Ca^2+^. With the exception of the caspases, structures of the inactive forms of other families in clan CD exhibit N- or C-terminal regions that block access to their active sites. It appears that the catalytic machinery in these enzymes is preformed, but the N- or C-terminal domain/regions sterically block substrate access, and a movement and/or cleavage in these regions is required for substrate binding.

### Activation mechanisms

#### Dimerization

The activation mechanisms for clan CD family members are reasonably diverse (see Table 2). One is dimerization. Activation by dimerization is required by several members of family C14, and the initiator and inflammatory caspases and MALT1 are all activated by this mechanism. In addition, the effector caspases are active only as dimers, although they exist in cells as inactive zymogens, so dimerization, although required, is not their activation mechanism. All the other family members of clan CD studied to date are active as monomers.

#### Internal processing

Another method of peptidase activation exhibited by clan CD enzymes is proteolysis. The effector caspases are activated by cleavage on L4–a loop region internal to the β-sheet, which is required for the correct formation of the active site. This cleavage became a defining structural feature of the archetypal members of clan CD, resulting in the caspases (along with the metacaspases and paracaspases) being (wrongly) described as composed of two subunits (large and small; before and after the cleavage site). Indeed, the effector caspases are the only group in clan CD for which cleavage of a loop region within the central β-sheet is required for activation or maturation; even structurally conserved MALT1-P and legumain have no known cleavage sites on L4 or within the peptidase domain. However, this is not to say that proteolysis is not important in the activation of clan CD enzymes: legumain, RgpB and MARTX-CPD all require cleavage, at sites external to their central β-sheets, for full activation to occur.

#### Ligand/pH change

Other activation mechanisms of clan CD enzymes include changes in pH (legumain) and the addition of ligands (Ins*P*_6_: MARTX-CPD, and Ca^2+^: metacaspases and RgpB). The pH change and allosteric binding of Ins*P*_6_ in legumain and MARTX-CPD, respectively, both result in movement around the sterically blocked active sites of the pro-forms which allows subsequent processing and/or substrate access. Less is known about the order of proteolysis and Ca^2+^ binding in RgpB, but there are no reports of enzyme activity with no Ca^2+^ present. It is interesting that the three Ca^2+^-binding sites identified in RgpB are not structurally conserved with the single site identified in TbMCA2. The location of two of the Ca^2+^-binding sites in RgpB suggests that they may contribute to the stability of the active site, although the third binds to residues on the protease that are involved in hydrogen bonding with the prodomain. The residues around the Ca^2+^-binding site in TbMCA2 are also involved in salt-bridge formation, with the N-terminal region, and it is intriguing to assume that Ca^2+^ could disrupt interactions of the inhibitory regions, producing (or allowing for) the conformational change required for substrate access.

## CONCLUSION

In conclusion, clan CD cysteine peptidases are a diverse group of enzymes found throughout the entire phylogenetic kingdom, exhibiting a wide range of functions, specificities and activation mechanisms. Structurally, they all contain a central β-sheet with a minimum of six strands (five parallel and one antiparallel) and are surrounded by various structural elements including a number of conserved α-helices. Substrate binding and specificity in the clan can be attributed to a few structurally homologous regions and the activity of many of the enzymes is self-regulated, to prevent undesirable proteolysis, through autoinhibitory mechanisms. The basic topology of the caspases (C14A), paracaspases (C14B) and legumain (C13) have been shown to be identical, whereas the topology of the metacaspases (C14B) is quite different, suggesting that metacaspases have been placed in the wrong structural family and adding to the opinion that metacaspases are not caspases [47,89] after all.

## Online data

Supplementary data
