# Supplementary material for: Comparative structural analysis of the caspase family with other clan CD cysteine peptidases
Source: Biochem J. 2015 Feb 20;466(Pt 2):219–32. doi: 10.1042/BJ20141324 (PMC4357240; doi:10.1042/BJ20141324)
Supplement: Supplementary data [file bj4660219ntsadd.pdf]

Supplementary Figure legends

FIGURE S1. Schematic diagram of the protein-ligand interactions in caspase-7. This structure was determined in complex with the inhibitor Ac-DEVD-CHO (Table S1). The inhibitor has a purple backbone and the interacting residues from caspase-7 are shown in orange. Carbon, oxygen, nitrogen and sulphur atoms are represented by black, blue, red and yellow spheres, resectively. The individual components of the inhibitor are denoted Ace701 (Ac)-Asp702-Glu703-Val704 (DEV)-Asj705 (D-CHO) and labelled in blue. Residues in caspase-7, which form hydrogen bonds to the inhibitor, are shown in green and those interacting specifically with the P_1 residue are underlined. Hydrophobic contacts with the ligand are represented by red semi-circles. This schematic was produced by LigPlot+ [26].

FIGURE S2. Primary sequences of TbMCA2 (PDB ID 4AFR) and Yca1 (PDB ID 4F6O) based on their structural alignment. Only the residues found in the crystal structures are present in this diagram. The SSEs of TbMCA2 and Yca1 are shown above the sequence in black and purple, respectively. The positions of the catalytic dyad, predicted ligand binding site residues and Ca^{2+} binding residues of TbMCA2 are shown in red (C/A mutation in TbMCA2), orange and cyan, respectively. Other identical residues that overlay in the structures are shown in black. The number of disordered N-terminal residues in TbMCA2 and Yca1 are reported in the titles (+3 and +90, respectively). The position of and number of disordered residues in the body of the structures are shown by an appropriately coloured down arrow (🡻) and delta (Δ) beside which the number of disordered residues are reported.

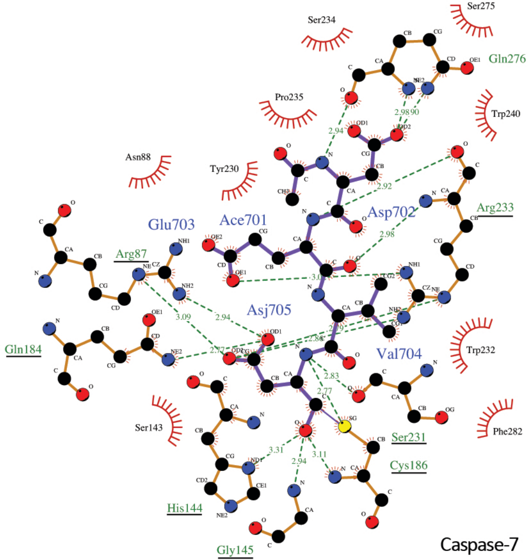
Ser275
Ser234
Gln276
Pro235
Trp240
Asn88
Tyr230
Arg233
Glu703
Ace701
Asp702
Arg87
Asj705
Gln184
Trp232
Val704
Ser143
Ser231
Phe282
Cys186
His144
Gly145
2.94
2.98
2.92
3.09
2.94
2.77
2.83
2.77
3.31
2.94
3.11

Caspase-7

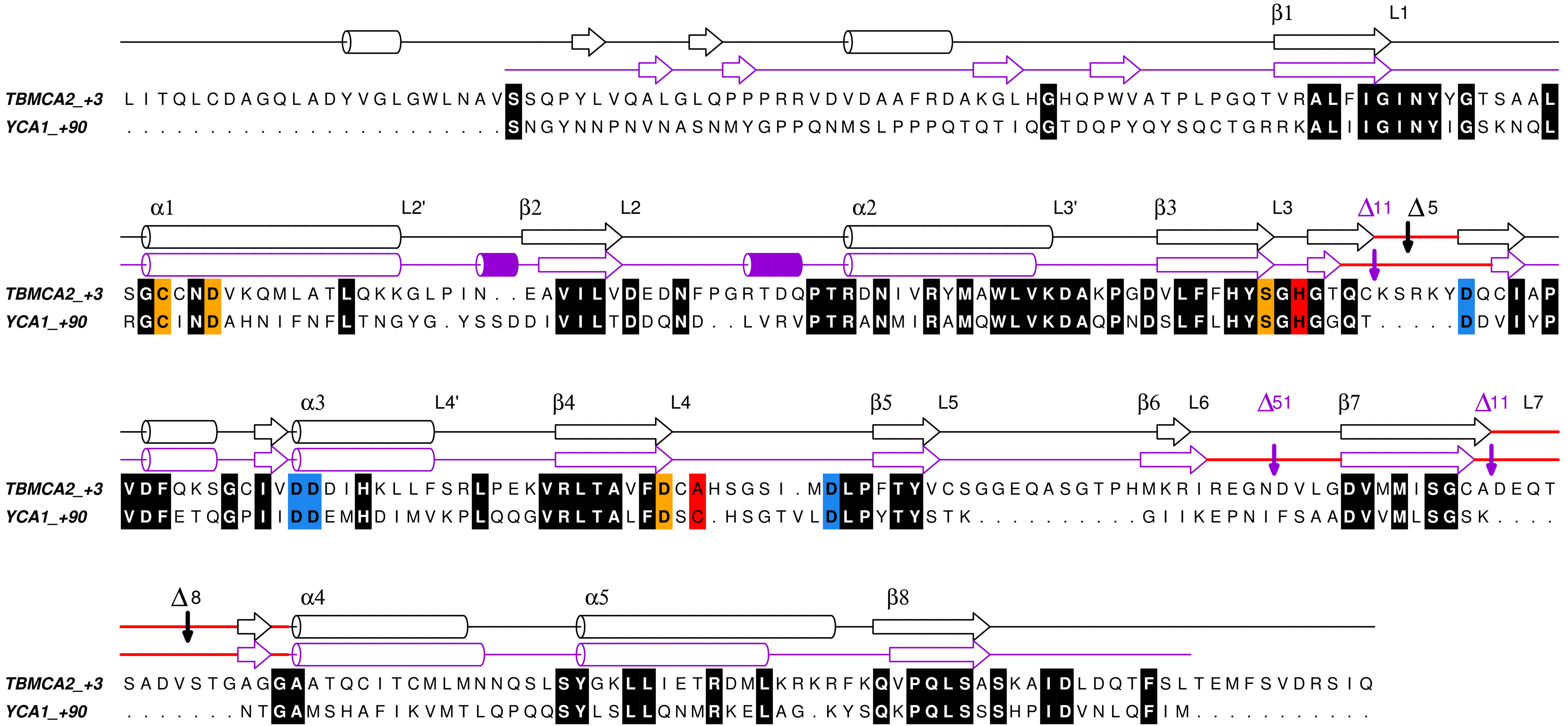

β1 L1
TBMCA2_+3 LITQLCDAGQLADYVGLGWLNAVSSQPYLVQALGLQPPPRRVDVDAAFRDAKGLHGHQPWVATPLPGQTVRALFIGINYYGTSAAL
YCA1_+90 ........................SNGYNNPNVNASNMYGPPQNMSLPPPQTQTIQGTDQPYQYSQCTGRRKALIIGINYIGSKNQL
α1 L2' β2 L2 α2 L3' β3 L3 Δ11 Δ5
TBMCA2_+3 SGCCNDVKQMLATLQKKGLPIN..EAVILVDEDNFPGRTDQPTRDNIVRYMAWLVKDAKPGDVLFFHYSGHGTQCKSRKYDQCIAP
YCA1_+90 RGCINDAHNIFNFLTNGYG.YSSDDIVILTDDQND..LVRVPTRANMIRAMQWLVKDAQPNDSLFLHYSGHGGQT.....DDVIYP
α3 L4' β4 L4 β5 L5 β6 L6 Δ51 β7 Δ11 L7
TBMCA2_+3 VDFQKSGCIVDDDIHKLLFSRLPEKVRLTAVFDCAHSGSI.MDLPFTYVCSGGEQASGTPHMKRIREGNDVLGDVMMISGCADEQT
YCA1_+90 VDFETQGPIIDDEMHDIMVKPLQQGVRLTALFDSC.HSGTVLDLPYTYSTK..........GIIKEPNIFSAADVVMLSGSK....
Δ8 α4 α5 β8
TBMCA2_+3 SADVSTGAGGAATQCITCMLMNNQSLSYGKLLIETRDMLKRKRFKQVPQLSASKAIDLDQTFSLTEMFSVDRSIQ
YCA1_+90 .......NTGAMSHAFIKVMTLQPQQSYLSLLQNMRKELAG.KYSQKPQLSSSHPIDVNLQFIM...........

Family: Enzyme name	PDB ID	State	Inhibitor/ligand
C11: PmC11	3UWS	Active, apo	–
C13: Legumain	4AW9	Active, inhibited, pH 7.5	Ac-YVAD-CMK
	4AWA	Active, inhibited, pH 5.0	Ac-YVAD-CMK
	4AWB	Active, inhibited	Z-Ala-Ala-AzaAsn-CMK
	4FGU	Proenzyme	–
C14A: Caspase-7	1F1J	Active, inhibited	Ac-DEVD-CHO
	1K88	Proenzyme	–
	1K86	Active, apo	–
	1I51	Active, inhibited	XIAP
C14B(P): MALT1-P	3V4O	Active, inhibited	Z-VRPR-FMK
	3UOA	Active plus Ig3 domain, inhibited	Z-VRPR-FMK
	3V55	Active plus Ig3 domain, apo	–
C14B(M): TbMCA2	4AFR	Inactive C213A, apo	–
	4AFV	Inactive, in presence of Ca^{2+}	–
	4AFP	Inactive, Sm^{3+} in Ca^{2+} binding site.	Sm^{3+}
C14B(M): Yca1	4F60	Active, apo.	–
C25: Gingipain R	1CVR	Active, inhibited.	D-FFR-CMK
	4IEF	Proenzyme.	–
C80: MARTX-CPD	3EEB	Active	$InsP_6$
	3GCD	Active, inhibited	Z-LLL-EP-COO-Et/ $InsP_6$
	3FZA	Proenzyme	$InsP_6$
C80:TcdA-CPD	3HO6	Active	$InsP_6$
C80:TcdB-CPD	3PEE	Active	$InsP_6$

Table S1: PDB codes for the crystal structures described in this manuscript, including the crystallisation state and any bound ligands and/or inhibitors present in the structures.

Enzyme	Family	Target		TbMCA2	Caspase-7	MALT1	Legumain	PmPNT1	MartX	Gingipain
Query		PDB ID		4AFR	1F1J	3V4O	4AW9	3UWS	3FZY	1CVR
TbMCA2	C14B(M)	4AFR		1.00	0.22	0.18	0.21	0.10	0.08	0.10
Caspase-7	C14	1F1J		0.22	1.00	0.41	0.34	0.14	0.10	0.13
MALT1-P	C14B(P)	3V4O		0.18	0.41	1.00	0.28	0.11	0.08	0.12
Legumain	C13	4AW9		0.21	0.34	0.28	1.00	0.13	0.11	0.12
PmC11	C11	3UWS		0.10	0.14	0.11	0.13	1.00	0.10	0.08
MARTX-CPD	C80	3FZY		0.08	0.11	0.08	0.11	0.10	1.00	0.05
Gingipain R	C25	1CVR		0.10	0.13	0.12	0.12	0.08	0.05	1.00

Table S2a: Structural homology of the structural families in clan CD. Each Query (column) was aligned with each Target (row) and the Q score (Q^S) calculated. The Q^S value for each Query is highlighted in grey. Where two values are the same caspase-7 is shaded slightly darker by default.

Enzyme	Family	Target		TbMCA2	Caspase-7	MALT1	Legumain	PmPNT1	MartX	Gingipain
Query		PDB ID		4AFR	1F1J	3V4O	4AW9	3UWS	3FZY	1CVR
TbMCA2	C14B(M)	4AFR		100	59	71	65	41	35	53
Caspase-7	C14A	1F1J		67	100	73	87	73	47	67
MALT1-P	C14B(P)	3V4O		86	79	100	79	71	50	64
Legumain	C13	4AW9		55	65	55	100	55	35	45
PmC11	C11	3UWS		24	38	34	38	100	24	28
MARTX-CPD	C80	3FZY		40	47	47	47	47	100	40
Gingipain R	C25	1CVR		30	32	29	29	26	19	100

Table S2b: Percentage of matched secondary structural elements (%SSEs) between all structural families in clan CD. The rows contain %SSEs of the Query identified in the Target and the columns contain %SSEs of the Target identified in the Query. The highest values for %SSEs are highlighted for each Target. Where two values are the same caspase-7 is shaded slightly darker by default.
